# A Virtual Sensor for Online Fault Detection of Multitooth-Tools

**DOI:** 10.3390/s110302773

**Published:** 2011-03-02

**Authors:** Andres Bustillo, Maritza Correa, Anibal Reñones

**Affiliations:** 1 Department of Civil Engineering, University of Burgos, C/Francisco de Vitoria s/n, 09006, Burgos, Spain; 2 Automatic and Robotic Centre (CAR), UPM-CSIC, Km. 0.200 La Poveda, Arganda del Rey, 28500, Madrid, Spain; E-Mail: maritza.correa@car.upm-csic.es; 3 CARTIF Foundation, Parque Tecnologico de Boecillo 205, 47151, Valladolid, Spain; E-Mail: aniren@cartif.es

**Keywords:** virtual sensor, Bayesian classifier, industrial applications, tool condition monitoring, multitooth-tools

## Abstract

The installation of suitable sensors close to the tool tip on milling centres is not possible in industrial environments. It is therefore necessary to design virtual sensors for these machines to perform online fault detection in many industrial tasks. This paper presents a virtual sensor for online fault detection of multitooth tools based on a Bayesian classifier. The device that performs this task applies mathematical models that function in conjunction with physical sensors. Only two experimental variables are collected from the milling centre that performs the machining operations: the electrical power consumption of the feed drive and the time required for machining each workpiece. The task of achieving reliable signals from a milling process is especially complex when multitooth tools are used, because each kind of cutting insert in the milling centre only works on each workpiece during a certain time window. Great effort has gone into designing a robust virtual sensor that can avoid re-calibration due to, e.g., maintenance operations. The virtual sensor developed as a result of this research is successfully validated under real conditions on a milling centre used for the mass production of automobile engine crankshafts. Recognition accuracy, calculated with a k-fold cross validation, had on average 0.957 of true positives and 0.986 of true negatives. Moreover, measured accuracy was 98%, which suggests that the virtual sensor correctly identifies new cases.

## 1. Improved Diagnosis of Faults in Multitooth Tool Machining: An Industrial Need

Automated procedures in which machine tools play a large part are well known in the manufacturing industry [1,2]. However, machine operators face complex decisions-making tasks to decide when to replace cutting tools due to wear [3]. Systems that monitor and evaluate cutting process are under constant development to enable the successful automation of machines. Key industries in the automobile and aviation sectors lead the demand for production line wear-detection procedures, which are invariably very difficult to implement in the real world.

These procedures continue to generate great interest in the research community [4], although only very few find their way into the industry itself [5]. Indeed, this question is the focus of the present study. A number of issues arise when working in industry, not least, when selecting suitable diagnostic systems and assessing the difficulty of placing a sensor in the required position for a given task. However, relevant information can be gathered which is helped by good production rates. Therefore, a lack of information from non-suitable sensors could be overcome by using intelligent virtual sensors that function with the knowledge obtained from the past behaviour of the milling machines. We consider a “virtual sensor” to be a device that estimates a product property by applying mathematical models in conjunction with information from physical sensors [6]. Virtual sensors collect and even replace data from physical sensors, in cases where their use is more convenient [7]. They are widely employed in such areas as mobile robotics (e.g., [8]) and have also been used in certain manufacturing processes [9,10].

The study presented in this paper focuses on the detection of insert breakage and overloads in a multitooth tool, which helps to eliminate deficient workpieces from the machining process, thereby avoiding irreversible damage. Overloads should be detected to inform the machine operator of changes in the cutting process that require timely analysis.

A number of business solutions exist which ensure that issues with the machine processes and cutting conditions are resolved. Of course, these processes require re-calibration so that negative alarms are reduced [11]. Re-calibration is important to limit negative alarms and maintain breakage detection rates at sufficiently high levels. The manufacturing industry sets out certain requirements for all devices that detect breakages:

- Sensors should be affordable and production rates need to be maintained.
- Fault detection has to be fast and reliable if it is to facilitate mass production. The diagnosis tool used to calculate breakage detection rate in machined workpieces is Mean Time to Detection (MTD). An MTD value of 2, for example, means the breakage rate is detected after the machining of 2 defected workpieces. Therefore an MTD value of 1 is optimum in real applications, which means that only one defected workpiece should be machined before detection of the breakage.
- The Mean Time between False Alarms (MTFA) needs to be increased, thereby avoiding false alarms which may occur because of spurious changes in the signals measured by the sensors. A diagnosis system should be able to detect a new breakage as soon as possible after an alarm. The diagnosis system is not usually available after an alarm for a certain number of workpieces, because it needs to collect information on the performance of the new cutting inserts before it generates a reliable diagnosis.
- Re-calibrations of the system should not be necessary.

The virtual sensors developed in this work consist of a system for the data acquisition of internal CNC signals, a module for signal processing and an intelligent decision-making scheme. The approaches that can be found in the literature for this task are mainly spectral analysis [12–14], wavelet transforms [15,16], fuzzy logic [17–19], neural networks [20–22], time domain processing [14,23,24] and hybrid systems [25,26]. In this paper, the time domain processing approach is considered, where a segmentation of the electrical power consumption takes place before a Bayesian network (BN) analysis is done to identify faults. The only variable under consideration is the electrical power consumption of the tool, because under industrial conditions other kinds of physical variables, such as acoustics or vibration signals, are not easily measured or are too noisy [27]. This new virtual sensor, which is based on power consumption analysis and Bayesian Networks classification-task capabilities, can be applied to different kinds of cutting operations. The proposed solution has been successfully applied to multitooth tools in the car industry under real conditions. Further applications of this technology are to be found in the mass production of metal pieces, including aluminium ribs for planes, and vehicle and lorry crankshafts.

The simplest Naïve Bayes [28] model is defined by the conjunction between the conditional independence hypothesis of the predictor variables (*X*_1_, . . ., *X_n_*) given the class (*C*) that represents the conditional probabilities *P* (*X_i_*|*C*); an independence case that is in reality quite rare. The joint probability density function for the networks is calculated by Equation 1, where *P* (*C_j_*|*X_i_*, . . ., *X_n_*) gives the probability that the discrete class variable *C* is in state *j*.

(1) $$P\left(C_{j}|X_{1},\ldots ,X_{n}\right)=P\left(C\right)\sum_{i=1}^{n}P\left(X_{i}|C\right)$$

This work uses the Tree Augmented Naïve Bayes (TAN) classifier [29], an extension of the Naïve Bayes model with a tree-like structure across the predictor variables. This tree is obtained by adapting the algorithm proposed by Chow & Liu [30] and calculating the conditional mutual information for each pair of variables given the class.

In recent years, Bayesian networks (BNs) have been used for fault diagnosis in industrial applications, for example, in an electric motor, as reported by [31]. The estimation of the *a priori* marginal and conditional probabilities for each node of the network were gleaned from expert knowledge. Different scenarios were proposed, which simulated damaged rotor blades, to identify vulnerable and critical components and to plan the appropriate maintenance tasks. In [32], a hybrid diagnosis system was proposed that combined sensor data and structural knowledge applied to the detection of broken rails that are part of railway infrastructure. Different neighbourhoods were selected to create 3 alternatives using a dynamic Bayesian network; however, the main problem with these solutions is that although the correct detection rate stands at about 99%, the false alarm rates were very high at 15%. In [33], a fault diagnosis was proposed for use in an industrial tank system. A BN was first obtained and then, a structure was defined as a Junction Tree. The results were compared with those obtained using polytrees, which in both cases yielded equally good results (about 60%) for simple faults.

Previously, an algorithm based on Linear Regression Outlier Detection had been used as a possible solution [23], which showed better results than CUSUM and time series forecasting. The CUSUM (CUmulative SUM of errors) is used to detect deviations of a signal from its mean value calculated by means of a RLS estimation with a forgetting factor. Multitooth tool behaviour is multi-faceted in the real world and requires experimental adjustment of a number of algorithmic parameters, for example, threshold levels. Finding a balance between false alarms and early detection of breakage was difficult to achieve. When 98% of breakages were detected, the MTD was 4.5 workpieces, whereas when the MTD fell to 2 workpieces the detected breakages were only 85%. Furthermore, a window of 70 workpieces should be considered to fit the algorithm after each breakage. This means, no breakage could be detected in the following 70 pieces after an alarm. This window is also necessary to improve the industrial performance of the diagnostic system. The aim of this work is to develop a new virtual sensor that radically enhances the MTD, keeps the number of false alarms as low as possible and reduces the fit window.

The paper is organised as follows. Section 2 contain a description of the virtual sensor. Section 3 introduces the experimental procedure for data collection including a discussion of sensor possibilities and the cutting process. Section 4 provides a detailed description of the results and compares them with previous works. Finally, in Section 5, the conclusions are presented and future lines of work are discussed.

## 2. Industrial Conditions and Virtual Sensor Description

In view of the specific task to be performed, a virtual sensor should be designed. It is therefore necessary to understand the special requirements of multitooth cutting and also the fault typology that should be detected before discussion of the design of the sensor.

### 2.1. Multitooth Fault Diagnosis

The term multitooth tool refers to many different types of tools. In this paper, the tools under analysis are used in the automobile industry for the mass production of the main journals and crankpins of automobile crankshafts. These tools are responsible for the initial roughing and finishing of the supports and crankpins of the workpiece. The workpiece material was cast iron, and due to the fact that it was the first operation there was an uncertainty or variation regarding the cutting forces between consecutive workpieces. This multitooth tool usually has an *ad-hoc* design for each crankshaft model and includes a large number of different cutting inserts. Each cutting insert is designed for a different operation: milling, broaching or turning and finishing or roughing. These tools are part of a mass production line and their parameterization is therefore fixed and only allows for minor adjustments (performed solely by the operator) to maintain a predefined geometrical tolerance. The tools that are analyzed in this paper are programmed in such a way that only cutting speed and feed may be adjusted. The cutting depth was fixed and predefined by the shape of the tool holder.

Most milling centres work with different tools in each cutting operation. The analysis of tool faults is easily performed as the milling centre records the tool that engages with the workpiece. A different diagnostics system may be implemented for each cutting operation. However, with multitooth tools the identification of the cutting insert group working at any one point in time is not so easy and requires a detailed temporal analysis of the signals from the selected sensors or signal segmentation. The segmentation can be formulated as the automatic decomposition of a signal into stationary or transient pieces with a length adapted to the local properties of the signal [34]. That is an important task in multitooth tool diagnosis prior to any diagnostic approaches.

Fault diagnosis in multitooth cutting means detection of breakages and overloads. Breakage means a sudden break of the cutting insert. Overload means a sudden power increase for a cutting operation due to minor manual adjustments in cutting conditions by the machine operator or for other unknown causes. The virtual sensor should detect these faults and warn the operator.

### 2.2. Virtual Sensor Description

**Figure 1.**
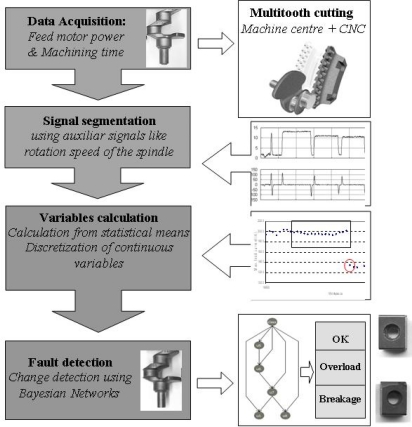
Scheme of the virtual sensor for multitooth fault detection.

The virtual sensor developed in this work consists of four modules: a data acquisition system capable of extracting information from the cutting process, a segmentation module to identify the information related to each group of cutting inserts, a third module to turn this information into different discretized variables and a final module to change the detection of the selected variables based on a Bayesian classifier for reliable multitooth fault detection. A schematic diagram of the virtual sensor is shown in Figure 1. Thus, Figure 1 also includes images of the crankshafts before and after the machining operation on the first body and images of broken and new cutting inserts for the roughing operation.

The first module is composed of the physical sensors that should provide information on the cutting process. There are several kinds of sensors that have been used in the literature for monitoring cutting processes: acoustic emission [35,36], cutting force [37,38], vibration [39,40], electrical power consumption [41,42] and noise [43,44]. In our case, a first attempt considered the temperature of the mechanized main journals of the crankshafts, vibration, noise and the electrical power of the tool drives as previously demonstrated in [23]. The electrical power consumption was measured from the output signals of the frequency converters of the two main milling machine motors driving the cutting process: the rotation motor and the feed motor showed the best signal-to-noise ratio. As this signal is also the least invasive, the cheapest, and remains unaffected by other events that might occur during machining [23], it was considered as the best physical signal for the virtual sensor. Between the two motors in which consumption is correlated with tool wear—the rotation motor and the feed motor, the latter showed a clearer correlation with tool faults [23]. The power consumption of the feed motor was therefore selected as the main input for the virtual sensor.

The second module is responsible for segmentation of the feed motor power consumption. The tool is divided into different groups of inserts, which also correlate with the temporal evolution of electrical power consumption. Each time a group of inserts engage the workpiece, the milling centre changes the cutting conditions (rotation speed and feedrate). The rotation of the tool is programmed in such a way that every group of inserts only ever engage each crankshaft once. Therefore, the segmentation of the electrical power signal for every insert uses tool rotation speed as an auxiliary signal to identify each group of inserts working at any given moment in time.

The third module generates discretized variables from the feed motor power consumption. Different statistical variables may be taken from this signal: maximum value, minimum value, mean value, integral of the whole interval, standard deviation, *etc.* Only the maximum of the feed motor power consumption for each machined workpiece is considered in order to compare the results of this paper with previous works using the same dataset [27]. Different variables should be calculated when machining other workpieces that provide as much information as possible on past tool behaviour. An important question concerns the best workpiece interval to consider? This interval is referred to as the Fit Window in this paper. A short Fit Window has to be considered because after every fault the Fit Window has to be restarted. Therefore, if the Fit Window is too long, many workpieces will have to be machined before the virtual sensor is ready to identify new faults. This interval was defined from the following estimate: if the breakage process were random, the probability of a new breakage before the virtual sensor is active (that is: the fit window size) would be below 50%. In the case of a Fit Window of 26 workpieces, this probability is 41.6% because the mean lifetime of the inserts is 1,000 workpieces and there are 16 types of insert. Hence, an interval of 26 workpieces was selected: the last machined workpiece and its former 25 workpieces. This interval is shorter than in previous works [23] where an interval of 40 to 100 workpieces was necessary for fault detection. Two assumptions are considered for this interval: feed power consumption evolution is almost negligible or feed power consumption shows a linear evolution. Both assumptions were considered to give flexibility to the detection model because different groups of inserts might behave in different ways. Up to 6 variables were then calculated, as detailed in Section 3.3, from the maximum power consumption for each workpiece considering both assumptions. After their calculation, the variables were discretized, because the analysis module only works with discretized variables. For this task, the discretization intervals were selected using data-independent criteria. The virtual sensor was therefore able to resolve them by itself for each insert group without the involvement of a human expert.

The last module predicts tool behaviour from the variables that were measured and discretized in the previous steps. This module uses a Bayesian classifier to select between 3 possible labels for the expected behaviour of the tool: type: “0” if the tool works properly, “−1” when a overload fault is presented and “+1” when a insert breakage takes place. The model is capable of detecting online faults in multitooth tools with probabilities of up to 90% for each fault type. Besides, the main advantage of BNs is their reasoning method, which is based on a model that attempts to convey the physical relationships of the process (milling in this case) and other less obvious (perhaps stochastic) relationships between the variables, not generally analysed in depth in other artificial intelligence-based models. This method in combination with the strong probabilistic theory of the BN generates their particular interpretations. The predictive capabilities of the virtual sensor will be explained in detail in Section 4.

## 3. Experimental Set-Up and Tuning of the Virtual Sensor to a Real Industrial Task

When the general schematic design of the virtual sensor was fully prepared, data from an industrial setting was collected to set up and to validate the virtual sensor.

### 3.1. Data Acquisition

Data were gathered from a multitooth tool that mechanizes the five main journals of a crankshaft. It includes 200 roughing inserts and around 30 finishing inserts. The tool manufacturer established insert life spans of around 1,100 workpieces in the case of the roughing inserts. During the machining cycle of each crankshaft, cutting conditions were varied according to a predefined profile. This profile relates to the kind of inserts (dedicated to roughing or finishing) that are spread over the tool’s surface and should work in each cycle instant.

**Figure 2.**
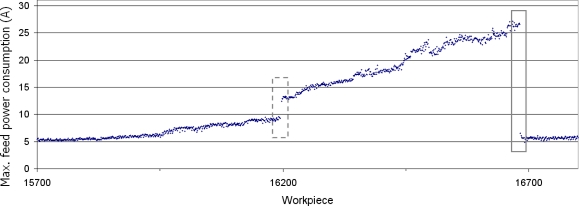
Evolution of maximum feed-power consumption in a group of roughing inserts over 1,000 machined workpieces.

Electrical power consumption is measured from the output signal of the frequency converter in the feed motor of the milling centre. The equipment used is based on a computer data acquisition system. An industrial PC with a data acquisition board that has a specific data acquisition software programmed in LabVIEW [45] v6.i monitors the different digital signals that handle the machining cycle. Each time a new workpiece starts its machining cycle, the software automatically performs data acquisition and recording. At the end of the cycle the software computes the diagnostics algorithms to determine whether anything is going wrong with the multitooth tool, so that the machine tool may if necessary be stopped to perform appropriate maintenance. As already explained, only the maximum of this power consumption for each machined workpiece is considered. An analysis of electrical power consumption during the cutting cycle shows how it rises slowly due to tool wear until insert breakage occurs, after which the corresponding power consumption waveform falls abruptly, as shown in Figure 2. This trend is clearly observable in the figure that shows the maximum feed-power consumption of a group of roughing inserts during the machining of over 1,000 crankshafts. The change instant due to tool breakage is marked by a grey square. This figure also shows overload as a grey dashed square, which signifies a small jump in the feed power consumption coming from minor modifications in cutting conditions manually performed by the machine operator or for other unknown reasons.

**Figure 3.**
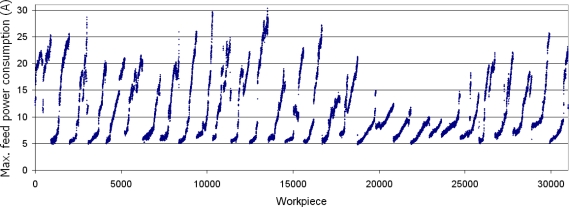
Data set used for the evaluation of the performance of the virtual sensor, representing the electrical power consumption of more than 30,000 mechanized crankshafts.

The entire dataset under analysis comprised over 30,000 mechanized crankshafts and included 57 insert breakages and 35 overloads, due mainly to manual changes in the cutting conditions by the machine operator. The dataset is shown in Figure 3.

### 3.2. Signal Segmentation

As the tool is divided into different groups of inserts, the temporal evolution of the electrical power consumption is also associated with the different groups of inserts. A signal segmentation of the temporal evolution of the electrical power consumption will facilitate identification of the electrical power consumption of each group of inserts each time a workpiece is machined.

The signal segmentation was carried out using *a priori* information such as drawings of the tool and the description of the machining cycle, mainly defined by cutting speed and depth. With these initial conditions, segmentation may be seen as a trivial process due to the repetitive nature of serial machining. However, there are external factors that differentiate each machining cycle from the point of view of its temporal evolution. The most important factors that can override the machining cycle are: manufacturing of more than one vehicle component on the same machine line, geometrical uncertainties of the component to be machined due to its casting or delays in the component supply line. All these reasons make it necessary to identify an algorithm that performs robust and reliable signal segmentation for further processing. A segmentation fault may lead to a false alarm that would affect the reliability of the virtual sensor.

**Figure 4.**
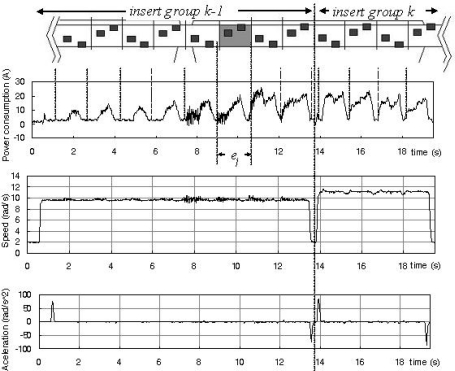
Signal segmentation based on the tool’s speed.

Among the different algorithms in use, those based solely on *a priori* assumptions (blind segmentation) or processing of the signal itself were shown to be ineffective. An auxiliary signal was therefore necessary to assure correct signal segmentation. The selected auxiliary signal was the tool’s speed. Figure 4 shows part of the machining cycle of the multitooth tool. The upper plot shows a partial drawing of the cutting tool that includes two insert groups and below the drawing, the electrical power consumption recorded by the data acquisition system of the virtual sensor. No pattern may be easily recognized with great certainty. A vertical line shows the change between two cutting insert groups. At the bottom, the tool’s acceleration and speed is plotted. Each insert group works at different tool speeds and a partial stop of the tool happens each time a new insert group moves into the cutting position. This pattern of acceleration peaks and different levels of constant speed may be used for signal segmentation.

**Figure 5.**
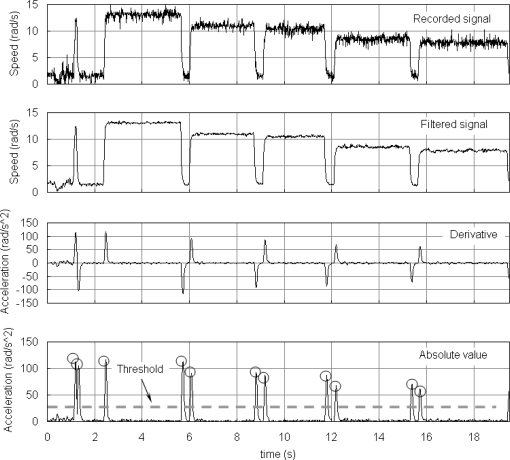
Processing stages of the tool speed signal.

Once the recognition pattern strategy is defined, the speed signal should be processed before the identification of cutting insert groups can take place. First of all, a moving average type filter is selected to reduce background noise. This filter keeps the sharp variations in the signal from tool starts and stops. Secondly, angular acceleration is calculated. To achieve a robust derivative, the acceleration signal is obtained from the slope of a moving linear regression of the speed. This technique reduces the noise in constant speed areas and emphasizes the changes that occur between cutting inserts change, which results in an auxiliary signal that refers to power consumption segments. Subsequently, the maximum absolute value of angular acceleration is then detected by means of a second-order polynomial fit. It is necessary to avoid the processing of constant speed areas to speed up the detection process of the maximums. A threshold for angular acceleration was therefore defined, below which the algorithm does not consider that a maximum could occur. Figure 5 shows an example of the successive stages of processing the tool’s speed signal.

### 3.3. Definition and Discretization of Input Variables

**Figure 6.**
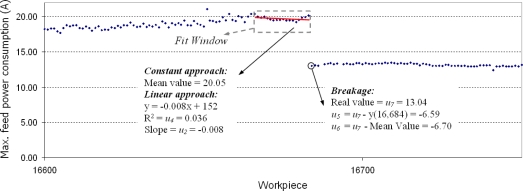
Two assumptions of electrical power consumption evolution: constant behaviour or linear evolution.

The maximum values are extracted from the signal segmentation of the electrical power consumption by the feed motor for each machined workpiece. From this value, 5 variables (described below) are calculated considering the behaviour of the tool in the 25 former pieces. Two assumptions may be made for this interval: either the feed power consumption evolution is almost negligible or the feed power consumption already shows a linear evolution. Both assumptions are considered in this work, to ensure that the virtual sensor is flexible, because different groups of inserts could behave in different ways. In the former case, the mean value of the power consumption of the former 25 workpieces is calculated. In the latter case, the power consumption linear fit of the former 25 workpieces and its correlation factor are calculated. Figure 6 shows both fits in one real case. The point under study is workpiece number 16,684, where a breakage occurs. The linear fit of the 25 former pieces predicts a feed power consumption of 19.63 A, and the mean value of the 25 former pieces is 19.74 A. In reality, however, a feed power consumption of 13.04 A occurs due to insert breakage. Figure 6 shows the Fit window, the linear fit and its main variables (slope and correlation factor), but also the change between real power consumption and expected power consumption for both approaches.

The following variables are then evaluated:

- *Time between last machined workpiece and present workpiece,* **u_1_**: this is the only variable that is not obtained from the feed power consumption, which includes information on machine stoppages that could be related to holidays, maintenance programs, manual adjustments made by the machine operator to cutting parameters, *etc.* These events can produce changes in the feed power consumption that are unrelated to tool faults.
- *Slope of the linear fit for power consumption evolution,* **u_2_**: searches for strong slopes in feed power consumption that usually predict breakage, as already done in Figure 6.
- *Correlation factor for last 10 workpieces,* **u_3_**: evaluates reliability of a linear fit for power consumption evolution within a short range of workpieces.
- *Correlation factor for last 25 workpieces,* **u_4_**: evaluates reliability of a linear fit for power consumption evolution, as previously shown in Figure 6, within a long range of workpieces.
- *Difference between expected power consumption for new workpiece and real value considering power consumption linear evolution,* **u_5_**: searches for strong drops in feed power consumption considering power consumption evolution to be linear.
- *Difference between expected power consumption for new workpiece and real value considering power consumption constant evolution,* **u_6_**: searches for strong drops in feed power consumption considering power consumption evolution to be zero.
- *Feed power consumption maximum value for the workpiece,* **u_7_**: searches for very low values that occur when tool inserts are either already broken and no longer cut or are completely new.
- *Number of machined workpieces since last breakage,* **u_8_**: this is a further factor that should be considered, as tool inserts have a mean lifetime of 1,000 workpieces.

When the variables are calculated for each workpiece, they should be discretized, because the classification module based on discrete Bayesian networks only works with discretized values. The discretization intervals could also be used to tune the virtual sensor for each insert group. The following criteria were considered for the definition of the discretization intervals:

- The variable range should be split in 4–5 discretization intervals
- The discretization intervals should be as user-independent as possible. Therefore, homogeneous intervals should be considered in all possible cases.
- In case homogeneous intervals do not allow extraction of all the information that the variable could provide, the discretization criteria to be considered should be defined in such a way that the computer can generate the discretization intervals automatically.

Table 1 summarises the considered variables and shows the discretization intervals considered for each variable. It can be seen that all variables are discretized at less than 5 levels. Some of the variables, such as **u_3_**, **u_4_** and **u_8_** are discretized in homogeneous intervals. Other variables that have a variation range symmetric around 0, such as **u_2_**, **u_5_** and **u_6_**, were discretized in symmetric intervals of around 0 with shorter intervals close to this point to increase the sensibility of the virtual sensor. The third group is composed of two variables: **u_1_** and **u_7_**, which often show a value of 250 and 15, respectively. The intervals are defined in such a way that two homogeneous intervals cover the range from the minimum value of the variable up to the most frequently occurring value, and three homogeneous intervals cover the range from this value to the maximum value. Thus, discretization may be automatically completed by the virtual sensor for each insert group and can clearly separate the behavior associated with the most frequently occurring value.

### 3.4. Bayesian Classifier

A dataset of 9 columns and 377 rows was formed to build a multitooth tool fault detection model for the virtual sensor. Each column of the dataset is a variable (the last is the class or network output) and each row is a machined workpiece. From the 377 machined workpieces, 285 showed no faults, 57 presented insert breakages and 35 overloads.

Before building the network of the Bayesian classifier a feature subset selection was performed to discard irrelevant variables. The goodness of a subset of variables was assessed by using a filter approach, *i.e.*, ranking the variables in terms of some scoring metric usually based on intrinsic characteristics of the data computed from simple statistics on their empirical distribution. Here, information gain is selected with regard to class variables as the scoring metric to evaluate the value of a variable. Information gain *I*(*class X*) is the difference between the entropy of *class* and the conditional entropy of *class* given *X* for any variable *X*. Weka [46] software, a free suite of machine learning software written in Java, developed at the University of Waikato (New Zealand), was used for this network learning process. Different structures were tested to build the BN structure that achieves the highest breakage detection rate. The best structure was identified when only 5 variables were selected: **u_5_**, **u_6_**, **u_7_**, **u_1_** and **u_8_**. The accuracy of this network was 98% compared, for example, with the figure of 97% for the network that incorporates all 8 variables.

**Figure 7.**
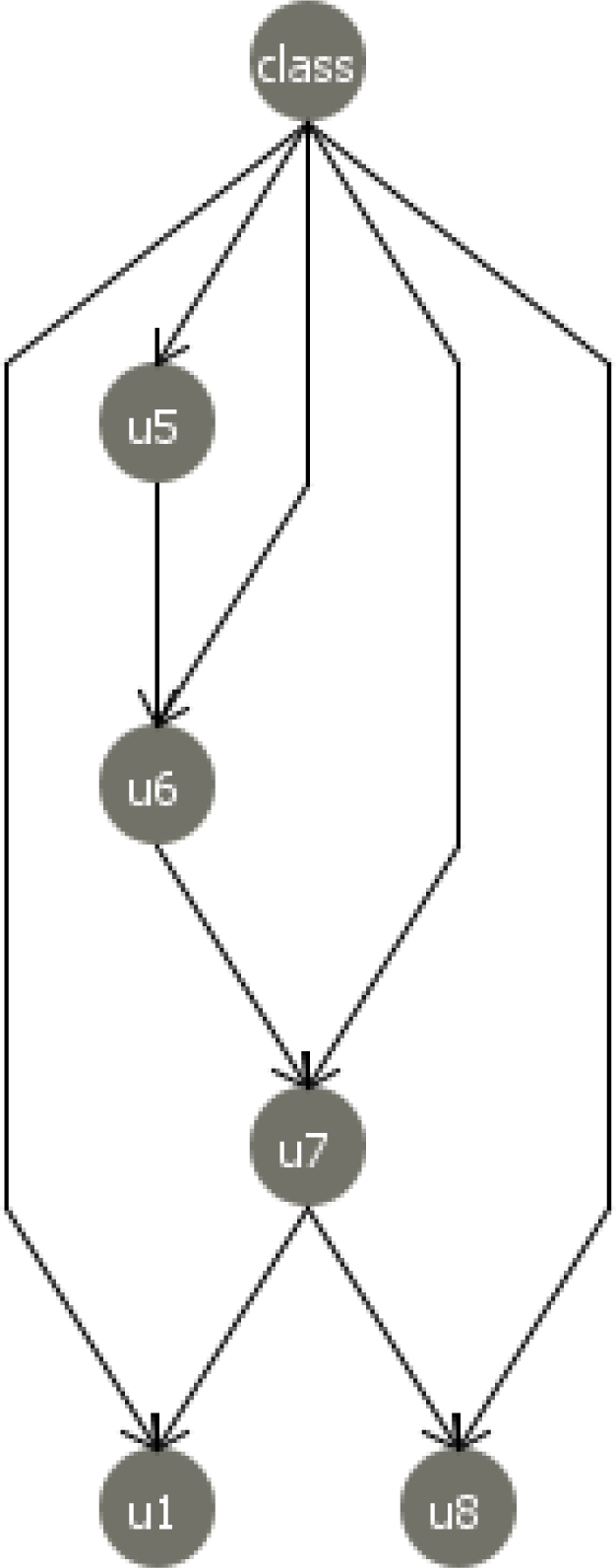
Bayesian network TAN structure.

Figure 7 shows the TAN structure built with the 5 selected variables. It illustrates the relationships existing between its nodes, which provide further information on the relationship of each variable with the class and on the relationship between all the predictor variables. The TAN network that is built also involves estimating all the conditional probability distributions of each variable given its parents, that is to say, its quantitative part. Given certain evidence (*i.e.*, observations), we can reason in any direction from any BN, querying the network about any marginal probability or any posterior probability .

Analyzing the network structure shows that the two variables **u_5_** and **u_6_** have the greatest influence on the fault type detected by the model. This relation was expected, as these variables collect information on sudden changes of power consumption when a breakage or an overload occurs while machining a piece. These are therefore the variables that best detect and identify the fault type during the machining process: the class or output variable.

Variable **u_7_** measures power consumption for the workpiece. This value is very high in overloads and very low when a breakage occurs, so it also has an important relationship with the fault type.

On the other hand, the influence of variables **u_1_** and **u_8_** and their relation to power consumption **u_7_** may also be appreciated. In **u_1_**, information is provided on machine time between workpieces; a variable that reflects stoppages on the production line, maintenance adjustments, *etc.*, which especially influences overloads faults, which are not reflected in other variables. In **u_8_**, the number of machined parts before tool breakages is recorded, which is also related to the state of the tool and the fault type that is detected.

The next section details an analysis of the results obtained with the Bayesian classifier for fault detection in multitooth tools.

## 4. Results and Discussion

As is well known, the models should not be validated with the same data used for training, for which purpose the 10*-fold cross-validation* [47] method was used. The initial dataset is divided into 10 subsets, of which only one subset is retained as the validation data for testing the model, and the other 9 subsets are used as training data. The *cross-validation* process is repeated 10 times (the folds), with each of the 10 subsamples used exactly once as the validation data. Then, the 10 results from the folds are averaged (or otherwise combined) to produce a single estimate of the accuracy of the classifier.

The accuracy of a model that is constructed in this way is high (98%), where accuracy is defined as the number of correct predictions over the number of samples. Accuracy is the most commonly used metric to evaluate the performance of a classifier, *i.e.*, an indicator of how good a classifier is or the probability of it classifying new cases correctly. But accuracy is not sufficient to determine the best model in this industrial process, because in this case the identification of tool breakage is more important than normal cutting behaviour. To clarify this point, consider a classifier that correctly identifies all the *Non Fault* cases but all *Breakages* cases are wrongly classified as overloads; this classifier will show a high accuracy, because there are very many more *Non Fault* cases than *Breakages* in the dataset, but the virtual sensor will be unable to identify the critical cases (tool breakage), which means that it would therefore be useless for industrial purposes. Other measures should also be defined and evaluated to assure correct classifier performance. To build these measures, a confusion matrix should be constructed that considers how many cases of each class *C_i_* (real) have been classified in each class *C_i_* (assigned), see Table 2. The following measures may then be considered: True Positive Rate (Sensitivity), True Negative Rate (Specificity), False Positive Rate and Precision.

Sensitivity or TPR (True Positive Rate) represents the proportion of items classified as belonging to class *C_i_*, from among everything that really belongs to class *C_i_*. In the confusion matrix (Table 2), it is the diagonal item divided by the sum of all elements of the row (Equation 2). When relevant sensitivities for each instance of class tends towards 1, the confusion matrix will tend to be a diagonal matrix.

(2) $$\begin{array}{ccc} \mathrm{TP}\;\mathrm{Rate}\;(\text{for 2 values}) & = & \frac{\mathrm{TP}}{\left(\mathrm{TP}+\mathrm{FN}\right)}\\ \mathrm{TP Rate}\left(C_{1}\right) & = & \frac{N_{11}}{N_{11}+N_{12}+\ldots +N_{1i}}\\ \vdots & & \\ \mathrm{TP Rate}\left(C_{i}\right) & = & \frac{N_{\mathrm{ii}}}{N_{i1}+N_{i2}+\ldots +N_{\mathrm{ii}}} \end{array}$$

False Positive Rate is the proportion of items that have been classified into class *C_i_*, but belong to a different class. In the confusion matrix, it is the sum of the column *C_i_* class minus the diagonal item, divided by the sum of the rows of all other classes (Equation 3).

(3) $$\begin{array}{ccc} \mathrm{FP Rate}\;(\text{for 2 values}) & = & \frac{\mathrm{FP}}{\left(\mathrm{FP}+\mathrm{TN}\right)}\\ \mathrm{FP Rate}\left(C_{1}\right) & = & \frac{N_{11}+N_{11}+\ldots +N_{i1}-N_{11}}{\left[\left(N_{21}+\ldots +N_{2i}\right)+\left(N_{31}+\ldots +N_{3i}\right)+\ldots +\left(N_{i1}+\ldots +N_{\mathrm{ii}}\right)\right]}\\ \vdots & & \\ \mathrm{FP Rate}\left(C_{i}\right) & = & \frac{N_{1i}+N_{2i}+\ldots +N_{\mathrm{ii}}-N_{\mathrm{ii}}}{\left[\left(N_{11}+\ldots +N_{1i}\right)+\left(N_{21}+\ldots +N_{2i}\right)+\ldots +\left(N_{(i-1)1}+\ldots +N_{(i-1)i}\right)\right]} \end{array}$$

Specificity or TNR (True Negative Rate) is the proportion of items that have been classified in other classes other than class *C_i_*. In the confusion matrix, it is the sum of the diagonals, minus the element of class *C_i_*, divided by the sum of the rows of the other classes (Equation 4).

(4) $$\begin{array}{ccc} \mathrm{TN Rate}\;(\text{for 2 values}) & = & 1-\mathrm{FPR}=1-\left[\frac{\mathrm{FP}}{\mathrm{FP}+\mathrm{TN}}\right]=\frac{\mathrm{TN}}{\mathrm{FP}+\mathrm{TN}}\\ \mathrm{TN Rate}\left(C_{1}\right) & = & \frac{N_{22}+N_{33}+\ldots +N_{\mathrm{ii}}}{\left(N_{21}+\ldots +N_{2i}\right)+\left(N_{31}+\ldots +N_{3i}\right)+\ldots +\left(N_{i1}+\ldots +N_{\mathrm{ii}}\right)}\\ \vdots & & \\ \mathrm{TN Rate}\left(C_{i}\right) & = & \frac{N_{11}+N_{22}+\ldots +N_{\left(i-1)(i-1\right)}}{\left(N-11+\ldots +N_{1i}\right)+\left(N_{21}+\ldots +N_{2i}\right)+\ldots +\left(N_{(i-1)1}+\ldots +N_{\left(i-1)(i-1\right)}\right)} \end{array}$$

Precision is defined in terms of the proportion of items that should actually belong to class *C_i_* from among all the elements that have been classified into class *C_i_*. In the confusion matrix it is the diagonal item, divided by the sum of the column under consideration (Equation 5).

(5) $$\begin{array}{ccc} \mathrm{Prec}(\text{Model}) & = & \frac{N_{11}+N_{22}+\ldots +N_{\mathrm{ii}}}{\text{Total examples}}\\ \mathrm{Prec}\left(C_{1}\right) & = & \frac{N_{11}}{N_{11}+N_{12}+\ldots +N_{1i}}\\ \vdots & & \\ \mathrm{Prec}\left(C_{i}\right) & = & \frac{N_{\mathrm{ii}}}{N_{i1}+N_{i2}+\ldots +N_{\mathrm{ii}}} \end{array}$$

In our case, Table 3 shows the confusion matrix generated by the Bayesian classifier and Table 4 summarises the three measures of merit for this classifier.

It can be concluded from Tables 3 and 4 that the sensitivity of the virtual sensor is 100% in the “breakage” class: all samples are correctly classified. This result is important in terms of the virtual sensor’s performance from an industrial point of view: all the breakages are detected when the first defective workpiece is machined and the rate of defective workpieces on the production line can clearly be reduced through the use of the virtual sensor. The False Positive Rate of this critical case is only 1.3% and therefore stoppage on the machining line due to false alarms for breakages is very low. In the case of overloads, the classification accuracy is lower, at 88.6%. This result is expected, because the overload class occurs less often in the dataset. This lower rate of overload detection does not matter from an industrial point of view, because overloads are not critical for tool performance or workpiece machining and the virtual sensor will only generate an alarm to notify the operator that something unexpected but not critical has occurred.

Comparison of the BN classifier with a former work on the same dataset [23] joins fault types, overloads and breakages in one category called “Fault” in Tables 3 and 4, the last row of which shows the classifier’s performance in the Fault class. In this case, the classification accuracy of the model is 96.4%. Other algorithms used to solve this industrial problem in a former work were the LROD algorithm, CUSUM and time series forecasting [23]. LROD showed the best capabilities to solve this problem. Therefore a comparison between LROD and the new virtual sensor was conducted. The LROD algorithm has three parameters to be fixed: the window size L, the fault’s threshold h and the run test parameter R. Different fits were tested [23] and their performance was compared with that of the new virtual sensor. The results are shown in Table 5. The objective searched for each LROD fit, which are also shown in this table. Performance is evaluated in terms of 4 criteria: MTFA, MTD, Fault classification accuracy and Fit Window.

It can be concluded that the LROD algorithm required a Fit Window of approximately 70 workpieces to achieve a percentage fault detection rate higher than 90%, while the virtual sensor required a Fit Window of only 26 workpieces to achieve a rate of 96.4%. If a shorter Fit Window is selected, for example 40 workpieces, the percentage of detected breakages for LROD drops to 88% in the best case. When a higher accuracy than 90% was sought, the LROD fit presented a MTD of more than 4.5 workpieces and a MTFA lower than 400 workpieces, which makes it an expensive solution because it would lead to many false alarms and unnecessary stoppages, and many defective workpieces would be machined. If MTD is decreased, LROD also looses precision and for a 2 workpiece MTD, only 85% of breakages can be detected. The virtual sensor presents an MTD of only 1 workpiece. The MTFA of the virtual sensor is not as good as some of the LROD fits, but its better performance in terms of accuracy and Fit Window with respect to these fits makes it the better solution. It should be noted that accuracy in this comparison was considered for the Fault class because former works do not split their results between Breakage and Overload classes, but the really critical class from the industrial point of view -Breakages- shows a true positive rate of 100% with the new virtual sensor.

In addition to its better overall performance, the new virtual sensor based on the Bayesian model presents a further advantage over the LROD algorithm: its reasoning capability. Given certain evidence, the BN may be queried on issues including observations or evidence, in order to find the *posterior* probability of any variable or set of variables. This can produce different types of reasoning.

One type of question is predictive reasoning or causal inference. For example, the question may be asked: what is the probability of each *class* (fault type) given certain manufacturing requirements? The response is a prediction of effects. If a batch of workpieces of between [800, 1,200] units with a maximum feed power consumption value has to be produced, the BN may be asked to compute *P* (*class|u*_8_ = 4, *u*_7_ = 3). Propagating this evidence, the network will compute the following class probabilities: non-fault with a probability of 0.46, Insert breakage with a probability of 0.33 and Overload fault with a probability of 0.21. Given these requirements, the highest probabilities are for non faults; which fits with the experimental dataset.

Questions relating to diagnostic reasoning may also be asked, such as “what are the probabilities of unobserved variables if class is restricted?” Let us suppose that we need to detect when a failure tool breakage will cause (class = Insert breakage) at the time of manufacturing a batch of workpieces (**u_8_**) between [400, 600), and that we need to establish the recommendations of the model with regard to maximum feed power consumption (**u_7_**) and machine time between workpieces (**u_1_**), so as to detect that type of tool breakage; in which case *P* (*u*_7_, *u*_1_*|class* = *insert breakage, u*_8_ = [400, 600)) is calculated. Moreover, a further advantage of BNs is the possibility of finding the most likely explanation or abductive inference. In this case, we look for the configurations of these two variables with the highest probability. The network informs us that the probability that this type of fault will occur increases as both machine time between workpieces and the maximum feed power consumption increase.

A total abduction finds the configuration of all the unobserved variables that maximize the evidence probability, e.g., *argmax_x_*_1_,...,*x_n_* *P* (*x*_1_, . . ., *x_n_*|*class* = *insert breakage*). In this case, the most likely configuration is **u_5_** at [−4, −0.5), **u_6_** at [−4, −0.5), **u_7_** at [0, 8), **u_1_** at 3 [800, 1,200] and **u_8_** at 4 [500, 10,000]. Thus, the most likely configuration of the remaining five variables responds to the question that concerns the conditions which have a higher probability of causing an insert breakage fault.

Another way to understand the usefulness of this capability of the virtual sensor is, for example, to schedule preventive tool changes. In this case, RB introduced evidence in two variables: (i) number of machined workpieces **u_8_** and (ii) feed power consumption maximum **u_7_**. The assumptions are normal machining with low feed power consumption (**u_7_** = 0) for the manufacture of 350 pieces (**u_8_** = 1), where the probability of breakage insert and overload fault is very low (0.062 and 0.038 respectively). The probability of insert breakages when the number of workpieces are increased may be calculated as follows: *P* (*class|u*_7_ = 0, *u*_8_ = 2). In this case, the probability of insert breakage and faults increases slightly without undue cause for alarm (0.128 and 0.079 respectively). However, the increased probability is considerable when *P* (*class|u*_7_ = 0, *u*_8_ = 3) is calculated: breakage 0.407 and faults 0.251, in the case of *P* (*class|u*_7_ = 0, *u*_8_ = 4) there is an increased probability of breakage at 0.56 and a fault probability at 0.346. From these results, we see that it is essential to change the tool when over 800 units produced.

## 5. Conclusions and Futures Lines of Work

This paper has presented a new virtual sensor for multitooth tool fault detection in the automotive industry. The proposed virtual sensor is based on electrical power consumption analysis and a Bayesian network. The application to a real case, the final machining of vehicle crankshafts on a mass production line was also presented and compared with a parallel methodology using the Linear Regression Outlier Detection Algorithm. The virtual sensor is defined as an alternative to the allocation of a suitable sensors close to the tool tip on the milling centres, because this strategy is not possible in industrial environments. The virtual sensor definition has to be capable of solving the problems that may occur with multitooth tools, especially complex machining tools with different kinds of inserts that perform different consecutive cutting operations on the same workpiece.

The virtual sensor consists of four modules: a data acquisition system capable of extracting information from the cutting process, a segmentation module to identify the information related to each kind of cutting inserts, a third module to turn this information in different discretized variables and a final module to change detection of the selected variables based on Bayesian classifiers in a way that is reliable for multitooth fault detection.

The virtual sensor detects faults through changes in electrical power consumption. As the tool is divided into different groups of inserts, the temporal evolution of the electrical power consumption is also associated with the different groups of inserts. The rotation speed of the machine spindle is used to identify the right time window for each insert group because the programmed rotation speed for each insert group is different.

As the electrical power consumption undergoes an abrupt change when a tool fault occurs, the problem of fault detection in a multitooth tool could be reformulated as a detection problem that uses different statistical variables of the electrical power consumption associated with the different groups of tool inserts, most of which consider the behaviour of the same tool during the machining of the former 25 workpieces. When the variables are calculated for each workpiece, they should be discretized because the Bayesian networks module only works with discretized values. The discretization intervals could also be used to tune the virtual sensor for each insert group. The discretization intervals were calculated using data-independent criteria. Therefore the virtual sensor could fix them by itself for each insert group without the interaction of a human expert.

The effective communication of an alarm after cutting operations on a workpiece must be made in a reliable manner, applying consistency checks and auxiliary information (cutting parameter changes, planned maintenance operations, *etc.*) for the process. This paper is mainly devoted to change detection and decision-making using a Bayesian model; an alternative that has mainly been chosen because of its potential to track and explain the process, which was tuned with the aid of different performance measurements. The interpretation capabilities of Bayesian networks allow the use of the virtual sensor not only as a fault detection sensor, but also as a predictive tool for machine line maintenance.

The new virtual sensor overcomes important drawbacks of the former proposed methodology based on the Linear Regression Outlier Detection Algorithm. The first is that the virtual sensor tuning is completely automated and does not depend on prior user knowledge of the cutting process. Thus, if a new multitooth tool is installed or the cutting process is changed, the auto-tuning capability of the virtual sensor can respond to this situation. The second it is that only a 26 workpiece interval before the workpiece that is machined in real time is necessary for fault detection. In previous works, a 40–70 workpiece interval was considered necessary to achieve high reliability. The new virtual sensor was therefore able to detect faults within a shorter period after the last fault. The third advantage is that the interval needed for fault detection with a high degree of reliability (higher than 95%) is reduced from the 4 workpieces cited in earlier works to 1 workpiece, which in turn drastically reduces defective workpieces produced on the machine line.

Future work will focus on the application of the virtual sensor to other kinds of industrial problems on which fault detection searches are conducted and a continuous degradation of certain variables is expected before fault detection occurs. Examples could be drill breakage during the drilling of multilayer sheets for the manufacture of aeronautical components, or mill breakages during the milling of aluminium ribs for aeroplanes.

## Figures and Tables

**Table 1.** Variables, units, values and discretization range.

Variable (Units)	Possible values	Range
*Time between last machined workpiece and present workpiece***u_1_***(seconds)*	0, 1, 2, 3, 4	[0,85), [85,250), [250,500),[500,10000), [10000,200000]
*Slope of the linear fit for power consumption evolution***u_2_***(A/workpiece)*	0, 1, 2, 3, 4	[−1.5,−0.15), [−0.15,−0.05), [−0.05,0.05), [0.05,0.15), [0.15,1.5]
*Correlation factor for last 10 workpieces***u_3_**	0, 1, 2, 3	[0,0.25), [0.25,0.5), [0.5,0.75), [0.75,1]
*Correlation factor for last 25 workpieces***u_4_**	0, 1, 2, 3	[0,0.25), [0.25,0.5), [0.5,0.75), [0.75,1]
*Difference between expected power consumption for new workpiece and real value considering a linear evolution of power consumption ,***u_5_***(A)*	0, 1, 2, 3, 4	[−4,−0.5), [−0.5,−0.15), [−0.15,0.15), [0.15,0.5), [0.5,4]
*Difference between expected power consumption for new workpiece and real value considering a constant evolution of power consumption***u_6_***(A)*	0, 1, 2, 3, 4	[−4,−0.5), [−0.5,−0.15), [−0.15,0.15), [−0.15,0.5), [0.5,4]
*Maximum feed power consumption value for the workpiece***u_7_***(A)*	0, 1, 2, 3	[0,8), [8,15), [15, 25), [25, 35]
*Number of machined workpieces since last breakage***u_8_**	0, 1, 2, 3, 4	[0,200), [200, 400), [400, 600), [600, 800), [800, 1200]
*Type of fault in multitooth tool* (class variable)	−1, 0, +1	−1 = overload fault 0 = non fault, normal cutting behaviour +1 = insert breakage

**Table 2.** Confusion matrix for more than 2 classes.

(category *i*) Assigned → Real ↓	*C*_1_	*C*_2_	. . .	*C_i_*
*C*_1_	*N*_11_	*N*_12_	. . .	*N*_1_*_i_*
*C*_2_	*N*_21_	*N*_22_	. . .	*N*_2_*_i_*
. . .	. . .	. . .		. . .
*C_i_*	*N_i_*_1_	*N_i_*_2_	. . .	*N_ii_*

**Table 3.** Confusion Matrix of Bayesian classifier.

Assigned → Real ↓	Non fault	Breakage	Overload
Non fault	281	4	0
Breakage	0	57	0
Overload	4	0	31
Fault (Breakage + Overload)	0 + 4 = 4	57 + 31 = 88	

**Table 4.** Accuracy by class of Bayesian classifier.

Class	TP Rate	FP Rate	Precision
Non fault	0.986	0.043	0.986
Breakage	1	0.013	0.934
Overload	0.886	0	1
Fault	0.957	0.014	0.957

**Table 5.** Comparison of the performance of LROD algorithm and classifier ensembles for imbalanced datasets.

	Linear Regression Outlier Detection				New Virtual Sensor
Parameters	R = 2, h = 4	R = 3, h = 2.5	R = 2, h = 1.1	R = 4, h = 3.5	
Criteria	Minimum MTD	Trade-off point	Maximum %Detected	Shorter Fit Window	
MTFA	2000	400	50	1000	463
MTD	2	6	4.5	3.5	1
%Detected	85%	93%	98%	88%	96.4%
Fit Window	70	70	70	40	25
